# Oxidative Stress and Liver Cancer: Etiology and Therapeutic Targets

**DOI:** 10.1155/2016/7891574

**Published:** 2016-11-10

**Authors:** Zhanpeng Wang, Zhuonan Li, Yanshuo Ye, Lijuan Xie, Wei Li

**Affiliations:** ^1^Department of Hepatobiliary-Pancreatic Surgery, China-Japan Union Hospital of Jilin University, Changchun 130033, China; ^2^Department of Plastic Surgery, China-Japan Union Hospital of Jilin University, Changchun 130033, China; ^3^Department of Vascular Surgery, China-Japan Union Hospital of Jilin University, Changchun 130033, China

## Abstract

Accumulating evidence has indicated that oxidative stress (OS) is associated with the development of hepatocellular carcinoma (HCC). However, the mechanisms remain largely unknown. Normally, OS occurs when the body receives any danger signal—from either an internal or external source—and further induces DNA oxidative damage and abnormal protein expression, placing the body into a state of vulnerability to the development of various diseases such as cancer. There are many factors involved in liver carcinogenesis, including hepatitis B virus (HBV) and hepatitis C virus (HCV) infection, alcohol abuse, and nonalcoholic fatty liver disease (NAFLD). The relationship between OS and HCC has recently been attracting increasing attention. Therefore, elucidation of the impact of OS on the development of liver carcinogenesis is very important for the prevention and treatment of liver cancer. This review focuses mainly on the relationship between OS and the development of HCC from the perspective of cellular and molecular mechanisms and the etiology and therapeutic targets of HCC.

## 1. Introduction

Oxidative stress (OS) is a process whereby the body receives stimulation from harmful endogenous or exogenous factors. Free radicals, including reactive oxygen species (ROS) and reactive nitrogen species (RNS), which are common metabolic products of several oxidation-reduction (redox) reactions in the cells, are increased when OS occurs. OS also induces DNA oxidative damage and abnormal protein expression, placing the body into a state of vulnerability. This is closely related to the occurrence and development of various diseases such as diabetes, cancer, and cardiovascular and nervous system diseases [1, 2]. A better understanding of the mechanisms of OS on human illnesses is very important for disease prevention and treatment.

Hepatocellular carcinoma (HCC) is the most common type of hepatic malignant tumor worldwide. Liver cirrhosis is acknowledged as a main risk factor for HCC, and the association rate is high, at 80–90% [3]. Many factors are involved in liver carcinogenesis, including HBV and HCV infection, alcohol abuse, nonalcoholic fatty liver disease (NAFLD), aflatoxin B1, obesity, diabetes, dietary habits, and iron accumulation [4]. Few studies have been conducted on the role of OS in the development of HCC; however, the relationship between OS and the pathogenesis of liver cancer has been attracting increasing attention. This report will provide a review of OS and the development of liver cancer from the perspective of cellular and molecular mechanisms and the etiology and therapeutic targets of HCC.

## 2. Mechanisms of OS-Related Liver Cell Injury and Carcinogenesis

**Figure 1:**
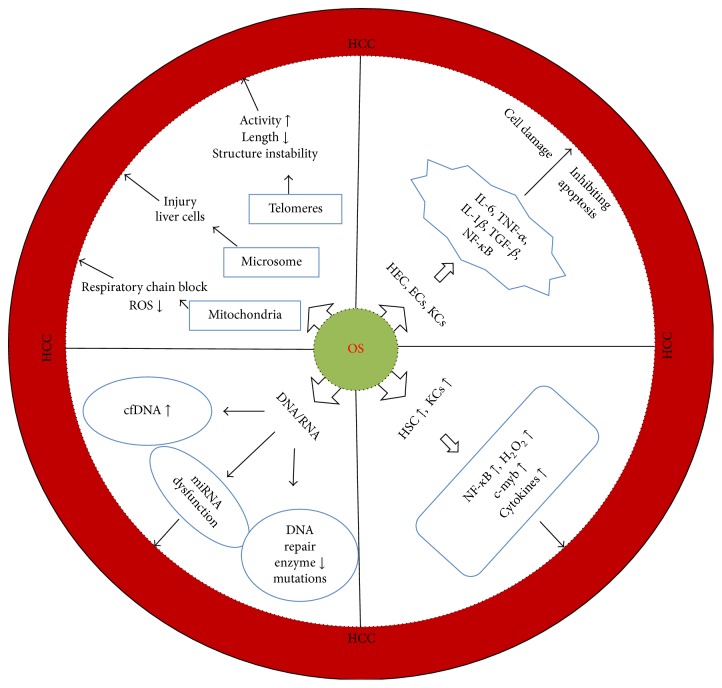
Mechanisms of oxidative stress on the regulation of liver cells.

In general, OS can be triggered by any dangerous or inflammatory signal and affects multiple cells in the liver. The mechanisms of OS on the development of liver cancer are summarized in Figure 1 and are described below.

### 2.1. Effects of OS on Cytokine Production and Cellular Apoptosis

Liver injury can be either an acute or a chronic inflammatory process. In the environment of local inflammation, many types of liver cells, such as liver sinusoidal endothelial cells (LSECs), hepatic stellate cells (HSCs), dendritic cells (DCs), and Kupffer cells (KCs), are activated. These cells produce many kinds of immune mediators, cytokines, and chemokines. For example, interleukin- (IL-) 6 is an important proinflammatory cytokine that can inhibit tissue inflammation and cellular apoptosis [5]. Tumor necrosis factor alpha (TNF-*α*) is a proinflammatory immune mediator that induces tissue damage, produces other cytokines, replenishes inflammatory cells, promotes the occurrence of fibrosis, and further activates the OS reaction [6]. One of the important functions of TNF-*α* is to activate cellular apoptotic and/or antiapoptotic pathways. The role of TNF-*α* in the development of HCC remains controversial [7]. OS-associated injury in chronic hepatitis patients is often associated with an increase in fibrosis factor TNF-*α* and transforming growth factor beta (TGF-*β*). TGF-*β* elevation is directly related to the severity of tissue injury and liver fibrosis [8].

Cytokines have been reported to affect liver inflammation, fibrosis, and apoptosis, regulate the process of alcoholic steatohepatitis (ASH)/nonalcoholic steatohepatitis (NASH), and participate in many metabolic changes of ASH/NASH, such as insulin resistance, lipid metabolism, appetite disorders, fever, and increased neutrophils [9].

### 2.2. OS and Mitochondria, Microsomes, and Telomeres

Mitochondrial dysfunction can impact many important cellular functions, leading to a variety of diseases [10]. New evidence shows that mitochondria play an important role in the process of carcinogenesis. During OS, mitochondrial transcription and replication are increased. The electron transport chain is blocked in the damaged mitochondria, resulting in accumulation of ROS. Further, TNF-*α* released by liver parenchymal cells and KCs directly damages the mitochondrial respiratory chain, consequently damaging mitochondrial cytochrome oxidase. On the other hand, the production of ROS is increased due to the blockade of any part of the respiratory chain; accumulation of ROS increases oxidative lipid deposition, which induces more lipid peroxidation, inhibiting the respiratory electron transport chain, creating a vicious circle [11]. Another vicious circle is the consumption of antioxidants. Fatty degeneration causes lipid peroxidation, and ROS can consume antioxidant enzymes, glutathione (GSH), and vitamin E; the loss of such protective material can enhance the effect of ROS on mitochondria [11]. Mass accumulation of ROS can change the mitochondrial metabolic process, increase the permeability of the mitochondrial membrane, promote the release of apoptotic factors, and further damage mitochondrial DNA and its additive effects of deletion and mutation [12]. The specific mechanism of signaling pathways becomes clear by illustrating how ROS and cancer-related proteins (p53, oncogenes) regulate mitochondrial functions [13].

Cytochrome P4502E1 (CYP2E1) is a microsomal oxygenase of fatty acid oxidation that can reduce the content of molecular oxygen and generate prooxidants. This process can lead to OS if it is not effectively blocked by an antioxidant. Administration of anti-CYP2E1 serum and a CYP2E1 inhibitor can block the process of OS and protect the cells from damage. In a human experimental liver NASH model, CYP2E1 surrounded the venules and was consistent with the most seriously damaged liver cells. All of these factors have proved that OS in microsomes can induce cell injury [14].

Telomeres play a very important role in cell proliferation, aging, immortalization, and carcinogenesis [15]. Telomere shortening may lead to an end-to-end fusion; consequently, somatic cells stop proliferating and enter into the stage of aging and apoptosis [16]. OS can accelerate the process of telomere shortening and speed up the accumulation of oxidative damage. In comparison with liver tissue from patients who have HCC with or without cirrhosis, HBV or HCV can induce changes in specific genes in the process of DNA repair, cell cycle control, and signal transduction of apoptosis (RASSF1A, GSTP1, CHRNA3, and DOK1 are specific genes that exist in HCC tumors) [17, 18]. According to recent reports, a chronic state of OS may cause migration of reverse transcriptase subunits of telomerase in the cytoplasm, thus reducing the activity of the enzyme. Reduction of the apoptosis signal in cells/tissue is a significant factor in carcinogenesis [19–21].

### 2.3. OS and Genetic Material

OS can cause DNA damage. One study showed that increased liver oxidative damage of DNA, combined with histological fibrosis, is a recognized risk factor for HCC [22]. Chronic viral infections cause liver cell necrosis and inflammation and liver regeneration, all of which are associated with infiltration of immune cells that produce reactive oxygen and nitrogen [23]. DNA damage induced by oxygen free radicals and DNA repair of the adaptation disorder leads to the accumulation of cancer-related gene mutations. There is much evidence that chronic inflammation is one of the causes of human cancer [24, 25]. Oxidative stress and accumulation of DNA damage play an important role in the process of virus-induced cancer [26]. The summary of HCC patients with oxidative DNA damage and inflammation markers was indicated in Table 1.

Circulating free DNA (cfDNA), which mainly comes from the oxide that DNA releases from dead cells, is a kind of DNA with double or single chain strands outside the cells. Circulating free DNA can be released by necrotic cells and apoptotic tumor cells. Low levels of cfDNA can also be detected in healthy people, but a higher level of cfDNA indicates the possibility of the presence of various diseases including cancer [27]. This phenomenon provides the basis for further research on the relationship between HCC and cfDNA.

Recently, microRNA (miRNA), a somewhat small noncoding RNA family (containing 21–23 nucleotides), has been found to play an important role in different phases of the process of HCC development [28]. In fact, miRNA inhibits the translation process by combining specific complementary sequences, or combining with specific complementary sequences on 3′UTR of mRNA to induce the degradation of mRNA [29]. miRNA is considered to be an important mediator in the immune system. Dysfunction of miRNA in inflammatory reactions and oncogenesis is the central event in the development of various cancers. When OS occurs, the expression of a variety of miRNA, as in HCC, is changed. The expression of miRNA-199a, miRNA-199b, and miRNA-122a in most (50%–70%) HCC is strongly downregulated, and the expression of miRNA-92 is indistinctively downregulated. On the contrary, the expression of miRNA-222 is upregulated [30].

### 2.4. OS and HSCs and KCs

In recent years, studies on the relationship between OS and HSCs have been increasing. HSCs have been proven to play a central part in the process of liver fibrosis [4]. HSCs can induce collagen production after activation in the body by free radicals, which are produced by ROS and superoxide anions, and further induce damage to liver cells [60, 61]. OS can further activate HSCs and stimulate the activity of nuclear factor kappa B (NF-kB). The NF-kB transcription factor is sensitive to redox. Activation of the NF-kB transcription factor can increase the production of nitric oxide (NO) and ROS, which participate in the formation of oxidized low-density lipoprotein (OxLDL) and further activate NF-kB. This creates a vicious circle and results in OS and cell injury [31].

KCs are liver macrophages that serve the functions of phagocytosis, antigen presentation, and immune regulation. KCs can be activated in response to danger of liver infection and produce various cytokines and inflammatory mediators, resulting in aggravation of liver cell injury [8]. Activated KCs produce a large amount of ROS and induce extracellular OS, which can directly cause liver cell necrosis. Other products of KCs, such as H_2_O_2_, NO, and various cytokines, may also have toxic effects on liver cells.

## 3. OS Potentiates Hepatitis Virus Infection and Liver Cell Carcinogenesis

**Figure 2:**
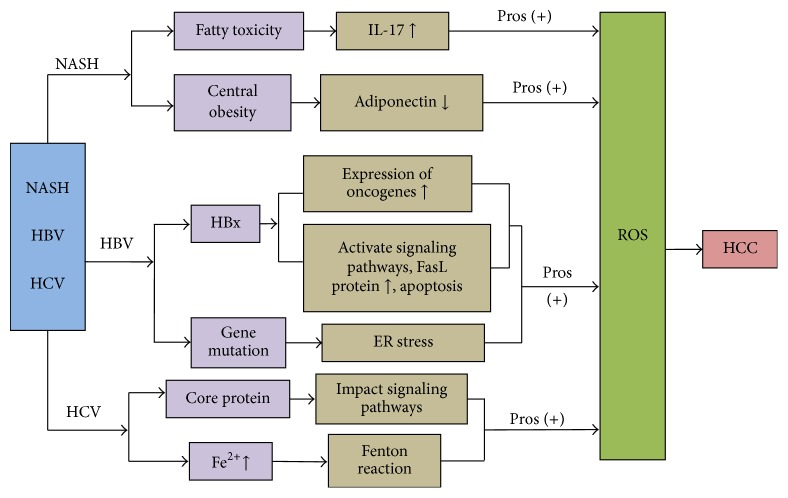
The mechanisms of oxidative stress on HBV-, HCV-, and NASH-related HCC.

It is known that over 80% of cases of HCC are associated with chronic HBV or HCV infection. Recently, the numbers of patients with obesity, as well as the related conditions of metabolic syndrome and NAFLD, are increasing, and these conditions are becoming an important cause of chronic liver disease in the developed countries, such as European nations and the United States. NAFLD includes simple fatty liver (SFL), nonalcoholic steatohepatitis (NASH), and related cirrhosis. NASH is also considered as one of the causes of liver cancer, and the mechanisms are under investigation. The mechanisms of OS in HBV-, HCV-, and NASH-related HCC are summarized in Figure 2 and are described below.

### 3.1. NASH-Related HCC and OS

The pathophysiological basis of NASH is a “two-hit” hypothesis. The first hit refers to the fatty degeneration of liver cells, characterized by the accumulation of triglyceride in the liver cells. The second hit includes a variety of cellular stress responses, such as apoptosis, OS, endoplasmic reticulum (ER) stress, and intestinal circumstances [62]. Other studies have demonstrated that the inflammatory response can induce fatty deposition in the liver cells, leading to the “multiple-hit” theory [63]. Fatty toxicity can cause multiple hits to the body, such as OS, ER stress, and immune responses [64]. Cellular stress is also involved in the process of carcinogenesis. The obesity-related diseases such as high blood triglycerides and high blood pressure are definite risk factors of NAFLD. OS is one of the important processes mediated by IL-17, while the IL-17 receptor is widely distributed on the surface of liver cells [32, 33]. The regulation of IL-17-related pathways has been shown to effectively prevent the development of NASH in a mouse model [34]. Patients with increased expression of serum IL-17 have a higher risk of early recurrence of liver cancer after surgery [35]. Thus, OS may be involved in IL-17-mediated NASH-related HCC.

Adiponectin is a protein from fat cells that regulates fat and carbohydrate metabolism [36]. In obese and diabetic patients, the level of adiponectin is usually decreased, and in patients with liver fibrosis, the level of adiponectin usually increases [37]. In HCC, the relationship between the adiponectin level and clinical features of the disease is very complex [38].

Most studies show that adiponectin is a “good” fat factor. Adiponectin has anti-inflammatory, antidiabetic, and anti-fat-accumulation properties; and it participates in energy metabolism, regulation of cell proliferation, and tissue remodeling [39, 40]. Adiponectin also inhibits the growth of cancer cells [41] and induces apoptosis [42], which is directly related to the occurrence and development of cancer [43]. Adiponectin inhibits angiogenesis and thus inhibits the growth and metastasis of liver tumors in mice [44]. Similarly, in a study of human HCC, a lower level of adiponectin has been found to be related to a higher malignant degree of HCC [45]. High adiponectin levels have been found to reduce the risk of prostate cancer, breast cancer, endometrial cancer, colorectal cancer [46], and pancreatic cancer [47]. Adiponectin blocks the protein expression of sulfatase 2 (SULF2), which is oncogenic, and high expression of SULF2 is related to HCC [48]. In addition, the expression levels of adiponectin in primary human liver cancer specimens are lower than in paracancerous tissues [49]. However, other studies have shown that adiponectin increases the risk of liver cancer. Aleksandrova et al. pointed out that non-high-molecular-weight (HMW) adiponectin, not high-molecular-weight adiponectin, was significantly associated with the risk of HCC [50]. Low-molecular forms of adiponectin are more closely associated with inflammation compared to high-molecular forms [51].

### 3.2. HBV/HCV-Related HCC and OS

HBV- and HCV-related chronic inflammation and fibrosis of the liver are usually induced by OS, which contributes to the pathogenesis of hepatocarcinogenesis. HBV infection results in activation of macrophages to produce a variety of proinflammatory cytokines, such as IL-1*β*, IL-6, CXCL-8, and TNF-*α* [52]. Such persistent abnormal production of cytokines and the resulting production of ROS have an influence on hepatocarcinogenesis.

The HBV genome can code a variety of gene products, including DNA polymerase (Pol), the capsid protein (core), envelope proteins L, M, and S, and the multifunctional protein HBx. Many studies have indicated that the HBx protein has carcinogenic potential. Transactivated HBx protein stimulates virus replication and expression and protects the virus-infected cells from damage [53]. The HBx protein is concentrated in the cytoplasm, and the c-terminal region from HBx's truncation is the producing region of ROS [54]. This phenomenon can be found in 46% of HCC tissue, but not in nonneoplastic tissue [55]. It is an important process in the development of liver cancer that HBV genes integrate into the host genome. Several cancer-related genes, such as TERT, MLL4, and CCNE1, can also be integrated by HBV [65]. HBx is the most common of these genes that are integrated into the human genome.

Studies on analyzing genetic mutations in HBV patients have found that these gene mutations were associated with the occurrence of liver cancer; this emphasizes the importance of HBx on the development of HCC, and OS is involved in this process. A considerable amount of experimental data has proved that the products from the mutant genes in the pre-s area, which accumulate in the ER, have the potential for promoting carcinogenesis through ER stress and the role of ROS [66].

All in all, OS, at least in part, participates in the process of HBV-related liver cancer development through HBx and the pre-s region.

In the state of HCV infection, liver antigen-presenting cells, KCs and DCs, are activated and modulate the immune functions [82]. The most direct impact of HCV on inflammatory signaling pathways is upregulating immunomodulatory molecules such as PD-L1 in KCs [56]. Persistent inflammation causes the liver cells to go into the circulation between the time of apoptosis and regeneration, produces a spontaneous mutation or damage to DNA, and further results in development of HCC [83]. HCV antigens, in particular the core protein, play a key role in the pathogenesis of chronic HCV and hepatocarcinogenesis through the TNFR, PKR, and STAT3 pathways [84]. There are more OS markers (8-OHdG) or reactive oxygen metabolites in the serum of HCV-related HCC patients than in HBV-related HCC patients, suggesting that there is more OS in HCV infection [57]. OS is also associated with senility, which is also one of the driving factors of hepatocarcinogenesis [85].

In addition, during chronic HCV infection, serological markers and iron accumulation in liver cells (especially in the lysosomes) usually are elevated. An excess of bivalent iron is strongly toxic due to induction of Fenton's reaction, ROS, and hydroxyl free radicals. Iron toxicity is considered to be one of the influencing factors of liver cancer. Some reports have shown that a diet low in iron can reduce the risk of hepatocarcinogenesis in patients with chronic HCV infection [58]. Additional research on iron metabolism and its correlation with liver cancer and OS is underway [59].

## 4. OS-Related Potential Therapeutic Targets

OS is associated with the development of HBV-, HCV-, and NASH-related HCC. Therefore, antioxidant treatment to control the causes of HCC is significant. In fact, there are many kinds of antioxidant drugs and foods in our everyday life. However, it is difficult to elucidate their specific effects in vivo and in vitro. Studies on the role of antioxidant effects on liver cancer development are still being conducted. The potential therapeutic targets on HCC of antioxidant treatment are summarized in Table 2.

It has been reported that curcuminoids could protect DNA from ROS damage, supporting the liver cells during the course of injury and cirrhosis [67]. Studies on chronic HCV infection have also shown that liver function was improved after antiviral treatment. Ascorbic acid, lipoic acid, and quercetin (types of flavonoid antioxidants) and mitoquinone (antioxidant agent target on mitochondria) are also beneficial to patients with chronic HCV infection. The antioxidant properties of resveratrol can reduce liver lipid peroxidation, increase the content of GSH in the liver, and scavenge ROS. The role of resveratrol is mainly in dealing with external liver damage factors such as alcohol intake. In addition, antioxidant drugs composed of ebselen (glutathione peroxidase analogue) have been used in early liver damage caused by alcohol. Studies have shown that vitamin E inhibits HBV replication and TGF-*β* gene expression in a rat model of NASH [68, 69].

Phlebotomy is considered an effective method against iron overload in patients with hepatitis and NASH [73]. Currently, alternative antioxidant treatment for liver cancer includes, but is not limited to, antioxidant gene therapy, induction of transcription factors AMPK or Nrf2 activator, and activation of oxygen scavengers and drugs that increase the capacity of mitochondrial oxygen intake. As antioxidant, the AMPK agonist metformin and mitochondrial support drug (L-carnitine) is more effective than vitamin E.

Metformin increases the level of AMP in the cells through the activation of AMPK and causes cell cycle arrest, apoptosis, and STAT3-induced IL-6 [74] and antioxidant enzyme of heme oxygenase-1 (HO-1) production [75] through the Nrf2 signaling pathway. A meta-analysis on the drugs of diabetes treatment found that the application of metformin can result in a 50% reduction in the incidence of HCC [76]. The use of 5-aminoimidazole-4-carboxamide-1-b-D-ribofuranoside (AICAR) as an AMPK activator can induce an increase in the Nrf2 protein and expression of antioxidant enzymes in endothelial cells, whereas AICAR activates Nrf2 in hepatoma cell lines resulting in antioxidant enzyme expression [77]. The combination of metformin and AICAR activates AMPK and Nrf2 for the purpose of controlling liver cancer. OS to normal cells can lead to a cancerous cell phenotype, in turn developing high resistance to further oxidative stress. At present, several clinical trials have found that the OS induced under these conditions can treat liver cancer [78]. The American Association for the Study of Liver Diseases (AASLD) recommends that the dose of vitamin E for the treatment of NASH should be 800 IU/d; in actual application, the dose administered is generally higher than the recommended dose [70]. The selection of the recommended dose was based on a two-year randomized study of NASH, which demonstrated that the dose can improve the level of alanine aminotransferase and histologic activity [71]. However, experimental studies found no beneficial effect on liver fibrosis [72]. Animal studies have shown that an L-carnitine dietary supplement can prevent chemically induced hepatitis and subsequent HCC and NASH-related HCC [79, 80]. Supplementation with L-carnitine was observed to significantly improve plasma glucose levels, lipid profiles, and histological manifestations of NASH patients [81].

## 5. Conclusion

**Figure 3:**
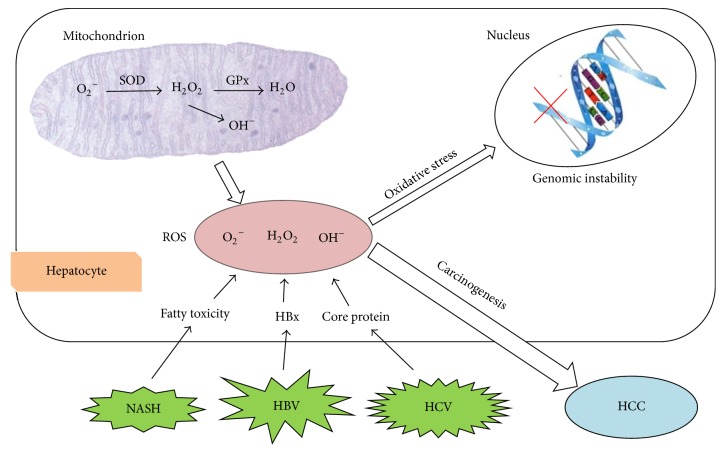
The mechanisms of OS-related HCC.

Accumulating evidence has shown that OS plays an important role in the development of liver cell carcinogenesis through disrupting either normal cell function or genetic materials and interfering with the pathways of signal transduction. Application of antioxidant drugs can control OS damage in vitro. However, so far there has been found no effective antioxidant drug that can be used in vivo. In order to design more effective methods for the prevention and treatment of HCC, investigations into better understanding the mechanisms of liver cancer development, OS damage, and antioxidants are urgently needed (Figure 3).

## Figures and Tables

**Table 1:** Summary of HCC patients with oxidative DNA damage and inflammation markers.

Ref number	Damage factors	Inflammation markers
[5]	HBV, HCV, NASH	IL-6
[6, 7]	HBV, HCV, NASH	TNF-*α*
[8]	HBV, HCV, NASH	TGF-*β*, H_2_O_2_, NO
[13]	Mitochondrial dysfunction	p53
[14]	NASH	CYP2E1
[17, 18]	HBV, HCV	RASSF1A, GSTP1, CHRNA3, DOK1
[27]	HBV, HCV, NASH	cfDNA
[28, 29]	HBV, HCV, NASH	miRNA
[30]	HBV, HCV, NASH	miRNA-199a, miR-199b, miR-122a, miR-92, miR-222
[31]	HBV, HCV, NASH	NF-kB, OxLDL
[32–35]	NASH	IL-17
[36–47]	NASH	Adiponectin
[48]	NASH	Sulfatase 2
[49–51]	NASH	Adiponectin
[52]	HBV	IL-1*β*, IL-6, CXCL-8, TNF-*α*
[53–55]	HBV	HBx
[56]	HCV	PD-L1
[57]	HBV, HCV	8-OHdG
[58, 59]	HCV	Fe^2+^

**Table 2:** Summary of antioxidant treatment targets in HCC therapy.

Ref number	Antioxidant treatment	Targets	Pros/cons HCC
[67]	Curcuminoids	Glutathione (GSH)↑, P450↓	Cons
[68, 69]	Ascorbic acid, lipoic acid quercetin, mitoquinone, ebselen	GSH	Cons
[68, 69]	Resveratrol	GSH↑, ROS	Cons
[70–72]	Vitamin E	HBV↓, TGF-*β*↓	Cons
[73]	Phlebotomy	Fe^2+^	Cons
[74–76]	Metformin	AMPK↑, Nrf2↑, IL-6↑, hemeoxygenase-1 (HO-1)↑	Cons
[77, 78]	5-Aminoimidazole-4-carboxamide-1-b-ribofuranoside (AICAR)	Nrf2↑	Cons
[79–81]	L-Carnitine	Mitochondria	Cons
